# Contaminant Metals and Cardiovascular Health

**DOI:** 10.3390/jcdd10110450

**Published:** 2023-10-31

**Authors:** Karl Kristian Lundin, Yusuf Kamran Qadeer, Zhen Wang, Salim Virani, Roman Leischik, Carl J. Lavie, Markus Strauss, Chayakrit Krittanawong

**Affiliations:** 1 Section of Cardiology, Baylor College of Medicine, Houston, TX 77030, USA; karl.lundin@bcm.edu (K.K.L.); yusuf.qadeer@bcm.edu (Y.K.Q.);; 2 Robert D. and Patricia E. Kern Center for the Science of Health Care Delivery, Mayo Clinic, Rochester, MN 55905, USA; 3 Division of Health Care Policy and Research, Department of Health Sciences Research, Mayo Clinic, Rochester, MN 55905, USA; 4 The Aga Khan University, Karachi 74800, Pakistan; 5 Section of Cardiology and Cardiovascular Research, Department of Medicine, Baylor College of Medicine, Houston, TX 77030, USA; 6 Department of Cardiology, Sector Preventive Medicine, Health Promotion, Faculty of Health, School of Medicine, University Witten/Herdecke, 58095 Hagen, Germany; 7 John Ochsner Heart and Vascular Institute, Ochsner Clinical School, The University of Queensland School of Medicine, New Orleans, LA 70121, USA; 8 Department of Cardiology I- Coronary and Periphal Vascular Disease, Heart Failure Medicine, University Hospital Muenster, Cardiol, 48149 Muenster, Germany; 9 Cardiology Division, NYU Langone Health and NYU School of Medicine, New York, NY 10016, USA

**Keywords:** cardiovascular disease, metal toxicity, metals and cardiovascular disease, cardiovascular toxicity, cardiovascular metal toxicity, heart and metal toxicity, environmental contaminants and cardiovascular toxicity, heart toxicity, cardiac toxicity, environmental heart toxins, environmental cardiac toxins, cardiology and metals, cardiology and environmental science, cardiology and metal toxins, heart toxins, cardiac toxins

## Abstract

A growing body of research has begun to link exposure to environmental contaminants, such as heavy metals, with a variety of negative health outcomes. In this paper, we sought to review the current research describing the impact of certain common contaminant metals on cardiovascular (CV) health. We reviewed ten metals: lead, barium, nickel, chromium, cadmium, arsenic, mercury, selenium, zinc, and copper. After a literature review, we briefly summarized the routes of environmental exposure, pathophysiological mechanisms, CV health impacts, and exposure prevention and/or mitigation strategies for each metal. The resulting article discloses a broad spectrum of pathological significance, from relatively benign substances with little to no described effects on CV health, such as chromium and selenium, to substances with a wide-ranging and relatively severe spectrum of CV pathologies, such as arsenic, cadmium, and lead. It is our hope that this article will provide clinicians with a practical overview of the impact of these common environmental contaminants on CV health as well as highlight areas that require further investigation to better understand how these metals impact the incidence and progression of CV diseases.

## 1. Introduction

Cardiovascular health, defined as the health or optimal functioning of the heart and blood vessels, is an essential aspect of human health, contributing significantly to both quality of life and lifespan. A growing body of research has begun to demonstrate the impact of environmental pollutants on cardiovascular health, with many environmental pollutants showing associations with the development of cardiovascular disease (CVD) and mortality. For example, particulate matter (PM) 2.5 exposure has been associated with an increased risk of CVD, CVD-related mortality, and all-cause mortality [1]. Modernization has brought human beings into increased contact with materials in forms and concentrations that were previously impossible. Among these, many contaminant metals brought into the human environment by new mining, manufacturing, and agricultural processes have been linked to a variety of negative health outcomes. There is growing evidence that exposure to contaminant metals may result in increased cardiovascular morbidity and mortality. In this review, we sought to examine 10 metals ubiquitous in the modern environment with wide-reaching and durable exposure to human populations across the globe. These metals are present in industrial processes, household products, or other novel routes of exposure that were not widespread before the modern era [see Table 1]. Our goal is to summarize which metals are most robustly implicated in CVD, explain the mechanisms by which they may cause CVD to arise, and engage in a brief discussion regarding public health measures needed to mitigate the negative impact of these metals.

## 2. Lead

Lead (Pb) is a natural part of our environment. In urban areas, lead is predominantly found in housepaint but is also found in the air due to the burning of gasoline with lead additives [2]. Lead exposure routes vary widely based on location; for example, lead exposure due to cosmetics and medications is common in India, whilst lead exposure through glazed ceramics and lead-contaminated utensils or water is more common in Mexico [3].

Multiple mechanisms have been proposed whereby lead may impact the cardiovascular system. For example, lead is implicated in the development of reactive oxygen species (ROS). In times of great oxidative stress, the production of excess ROS results in tissue damage and is thought to play a critical part in the development of CVD [4,5]. Gonick and colleagues showed that rats with lead-induced hypertension accumulated lipid peroxidation products and had increased amounts of inducible NO synthase enzymes [6]. Lead exposure also appears to have a role in causing vascular constriction through effects on protein kinase C (PKC). Protein Kinase C is involved in many cellular functions, including cell growth, vascular contraction, blood flow, permeability, and overall cell survival [7]. In an animal model, Watts and colleagues showed that lead caused the contraction of mesenteric arteries and further found that lead-induced contraction of those arteries was enhanced by a PKC agonist and reduced by a PKC inhibitor [8]. In addition, lead exposure activates the nuclear factor-kB (NF-kB) family of transcription factors, which has downstream effects including inflammation, apoptosis, and fibrosis [9]. Rodriguez-Itarbe and colleagues showed marked NF-kB activation, tubulointerstitial accumulation of T cells, macrophages, and angiotensin II-expressing cells, increased number of apoptotic cells, and heavy tyrosine nitration in kidneys of rats with lead-induced hypertension (HTN) [10]. Bravo and colleagues sought to suppress the inflammatory response in rats with lead-induced hypertension. They found that immunosuppression (via mycophenolate mofetil) prevented HTN, oxidative stress, and NF-κB activation, attenuated tubulointerstitial lymphocyte and macrophage infiltration, and reduced the number of angiotensin II-expressing cells in the lead-exposed animals [11]. 

Lead exposure is also implicated in hormonal alterations that may be harmful to cardiovascular health. Chang and colleagues found that in workers exposed to lead, there were higher levels of circulating plasma norepinephrine levels [12]. Likewise, Khalil-Manesh and colleagues found that rats exposed to low levels of lead had increased blood pressure and plasma endothelin-3 concentrations [13]. Endothelins are potent vasoconstrictors and have been implicated in CVD [14]. Lead exposure may impact the renin–angiotensinogen–aldosterone (RAAS) pathway as well, as evidenced by an animal model conducted by Vander and colleagues [15]. 

Studies conducted by Lustber and Schober linked increasing exposure to lead to an increased risk of CVD-related mortality [16,17]. Lustber and colleagues used the mortality follow-up data for participants of the Second National Health and Nutrition Examination Survey (NHANES), a national cross-sectional survey of the general population, conducted from 1976 to 1980. They followed 4292 participants aged 30 to 74 years with blood lead measurements through 31 December 1992. After adjustment for potential confounders, individuals with baseline blood lead levels of 20 to 29 microg/dL (1.0–1.4 micromol/L) had a 46% increase in all-cause mortality (rate ratio [RR], 1.46; 95% confidence interval [CI], 1.14–1.86), 39% increase in circulatory mortality (RR, 1.39; 95% CI, 1.01–1.91), and 68% increase in cancer mortality (RR, 1.68; 95% CI, 1.02–2.78) compared with those with blood lead levels of less than 10 microg/dL (<0.5 micromol/L). Muntner and Navas-Acien showed an association between blood lead and peripheral artery disease (PAD) [18,19]. Munter’s study utilized data from two nationally representative cross-sectional surveys, the Third National Health and Nutrition Examination Survey conducted in 1988–1994 (*n* = 16,609), and the National Health and Nutrition Examination Survey conducted in 1999–2002 (*n* = 9961). After multivariable adjustment, persons in the highest quartile (> or = 2.47 microg/dL [> or = 0.12 micromol/L]) compared with those in the lowest quartile (<1.06 microg/dL [<0.05 micromol/L]) of blood lead levels were 1.92 (95% CI, 1.02–3.61) times more likely to have peripheral arterial disease. Some studies show a positive association between higher blood lead levels and increased incidence of coronary artery disease (CAD) or stroke [20,21,22], though this may be attributable to confounding factors, such as cigarette exposure [20]. Also, lead may have a role in affecting the electrical conduction system of the heart [23]. As shown in a robust meta-analysis conducted by Nacas Acien and colleagues, the underlying basis for lead exposure and CVD seems to be its role in promoting hypertension, one of the strongest risk factors for development of CVD [24].

Given the wide-ranging and extensive associations between lead and CVD, it is imperative that prevention strategies are implemented to reduce the general population’s exposure to lead. The United States Environmental Protection Agency (EPA) has guidelines for regulating the amount of lead in air and water, but enforcing these levels has failed at times (as evidenced in the Flint, MI water crisis). Updating housing in areas that use lead-based paint will also serve to decrease exposure to lead.

## 3. Barium

Barium (Ba) is ubiquitous in nature in a water-insoluble state as either barium sulfate or barium carbonate [25]. Common exposures include ingestion of barium-containing materials and barium-contaminated water [26]. 

Barium toxicity was associated with gastrointestinal side effects as well as hypokalemia, ST changes, and ventricular extrasystoles in one series of case reports involving 39 cases and 226 human subjects [27]. Unfortunately, there have not been many studies investigating the role of Barium on CVD. One study conducted by Wones and colleagues investigated the role increasing levels of barium in water had on an individual’s risk factors for cardiovascular disease. They enrolled 11 men in a 10-week study where they increased the Barium in drinking water from 0 ppm to 5 ppm to 10 ppm. They found that there was no change in systolic or diastolic blood pressure, cholesterol, potassium, glucose, or urine catecholamine levels. There was a borderline statistically significant increase in serum calcium levels, but this was of uncertain clinical significance. Thus, at least from this study, barium did not seem to have much of an effect on the development of CVD risk factors [28]. However, in an animal model, exposure to increased concentrations of barium resulted in an increase in blood pressure and a decrease in cardiac contractility and electrical excitability in the heart [29]. A retrospective study conducted by Brenniman and colleagues investigated the differences between Illinois communities with high barium levels in water (2–10 mg/liter) compared to Illinois communities with low barium levels in the water. Results of this mortality study revealed that the high barium communities had significantly higher (*p* < 0.05) death rates for “all cardiovascular diseases” and “heart disease” compared to the low barium communities [30].

The EPA has recommended that barium concentrations not exceed 2 mg/liter of water [31]. We have not been able to locate studies showing that 2 mg/liter of water is a specific threshold for barium toxicity, but there is some evidence to suggest that higher levels of barium are detrimental. More studies are needed to determine the safe level of exposure to barium and guide the formation of public health measures to reduce exposure to toxic levels of this metal. 

## 4. Nickel

Human exposure to nickel (Ni) occurs primarily via contamination of drinking water and food; nickel is highly mobile in soil and is, therefore, able to readily contaminate water and food supplies [32]. Environmental pollution from nickel can also occur secondary to industrial processes, fuel burning, and inappropriate disposal of waste products [33]. 

Nickel seems to exert cardiotoxic effects through the generation of free radicals. Novelli and colleagues discovered that increased nickel exposure increased the lipoperoxide and lipid concentrations in the cardiac tissue of male Wistar rats, with the superoxide radical (O_2_^−^) being central to the cardiac damage [34]. 

A study conducted by Zhang and colleagues in China provided some evidence of an association between nickel exposure and congenital heart disease. Based on 490 controls and almost 400 cases, they were able to conclude that there seemed to be an association between nickel exposure and the development of congenital heart disease [35]. One proposed explanation is that nickel causes mutations in the mitotic apparatus, precipitating premature cell death during fetal development [36]. Nickel may also cause epigenetic alterations and/or produce reactive oxygen species [37,38]. A recent study published by Cheek and colleagues sought to address the impact of nickel exposure on cardiovascular disease. Using the National Health and Nutrition Examination Survey from 2017–2020, urinary nickel concentration was measured in individuals with a diagnosis of CVD. They found that independent of traditional CVD risk factors, nickel exposure was associated with CVD [39]. Nickel exposure was also shown to be related to the number of carotid arteries with plaques in a Swedish cross-sectional study [40]. Finally, a dose-dependent association between nickel exposure and hypertension was observed by Shi and colleagues in a study conducted in China [41]. In this study, they recruited 940 participants from six factories in northeastern China and measured the urinary concentrations of 19 metals. They then used Bayesian kernel machine regression (BKMR) to explore associations between metal co-exposure and hypertension. The BKMR model indicated a hermetic dose-response relationship between eight urinary metals (Cobalt (Co), Chromium (Cr), Ni, Cadmium (Cd), Arsenic (As), Iron (Fe), Zinc (Zn), and Pb) and hypertension risk. 

The Occupational Safety and Health Administration (OSHA) has set a guideline of 1 mg/m^3^ of nickel compounds in work-room air during an 8 h shift to protect workers. In addition, the EPA has set a guideline of 0.1 mg per liter of nickel in drinking water for the general public [42]. More robust data may be needed to validate these guidelines and determine the exact nickel levels associated with an increased risk of CVD. 

## 5. Chromium

Chromium is a transition metal that naturally exists in small amounts in plants, animals, and the environment. It is used in a wide number of industries, including leather tanning, textile dying, paint pigmentation, wood preservation, and metal plating. The waste from these industries is often used for filling material for dikes, marshland reclamation, and backfilling sites after demolition, allowing chromium to seep into water sources and contaminate the soil and food supply [43]. Nontoxic but elevated levels of airborne exposure have also been reported from those who frequently work in or travel via the New York City subway system, and respiratory toxicity due to occupational inhalation is well described [44,45]. 

Though chromium can exist in multiple oxidation states from Cr(-II) to Cr(+VI), Cr(III) and Cr (VI) are the most stable, prevalent, and biochemically relevant. Cr(III) is an essential nutrient required for the glucose tolerance factor (GTF), which is a cofactor that binds insulin to receptor sites on membranes. The exact mechanism is poorly understood, but Cr(III) has been shown to both increase insulin phosphokinase activity and decrease insulin phosphatase activity, thereby increasing insulin sensitivity [46,47]. Studies have demonstrated chromium deficiency’s association with increased risk of metabolic syndrome [48], diabetes and cardiovascular disease [49,50,51], and myocardial infarction [52], and chromium supplementation conversely has been shown to improve lipid profiles in human [53] and animal [53] studies. Some studies have even shown Cr(III) administration causes regression of atheromatous plaques in animal models [53,54].

However, Cr(VI) is a potent carcinogen, though any cardiovascular toxicities are not well reported [55,56]. Cr(VI) is able to easily enter cells through sulfate channels due to its structural similarity to sulfate. Once inside the cell, Cr(VI) is either rapidly reduced, a process which can generate free radical byproducts and increase oxidative stress, or it may bind to deoxyribonucleic acid (DNA) to form Cr-DNA adducts with high mutagenic potential. Gastrointestinal and respiratory cancers are the primary illnesses linked to Cr(VI) [56,57]. One study did describe some alterations in echocardiography, ballistocardiography, kinetocardiography, and rheocardiography in 230 workers with symptomatic occupation chromium exposure compared to 70 healthy controls [57], and a case report of toxic chromate–copper–arsenate ingestion described cardiovascular complications though any inferences are difficult to draw due to the presence of several other toxic compounds in the ingestion [58]. One recent study has shown Cr(VI) induces apoptosis and autophagy of cardiomyocytes from broiler chickens in a dose-dependent manner via oxidative stress and the induction of mitochondrial dysfunction [59].

Diagnostic studies used to measure chromium levels include serum [49,51] and levels in toenail clippings [52]. Improved occupational safety standards and reduction in dumping of chromium-tainted waste are the primary avenues to reduce exposure. Novel techniques, such as using a “soil-plant” barrier, where plants that reduce highly toxic Cr(VI) into the safer Cr (III) are planted near dumping sites, have also been proposed [43].

## 6. Cadmium

Cadmium is a naturally occurring metal found in zinc and lead ores, as well as phosphate fertilizers [60], and is often ingested in contaminated food and water [61] or through occupational exposures related to the smelting, battery, or pigment production industries [62,63]. Cigarette smoke is also a significant source of cadmium exposure, partly due to the tobacco plant’s high affinity for this metal [60,61,62,63]. Cadmium exposure often varies greatly by region; for example, exposure levels in certain polluted areas of Thailand have been recorded at roughly 20 times higher than exposure levels in some areas of Sweden [60,61].

Cadmium is thought to exert damaging effects on both myocardial and vascular endothelial cells through a variety of mechanisms, as well as contributing to the development of comorbidities often implicated in the onset and exacerbation of CVD (e.g., hypertension) [64]. The exact mechanism for cadmium entry into cardiac myocytes and endothelial cells is still under investigation; there is evidence implicating both the divalent metal transporter (DMT) and endocytosis as the mechanisms responsible for the entry of the cadmium into other types of cells in its ionized and protein-bound forms respectively [65,66]. Cadmium may also enter endothelium via infiltrating immune cells, which have been shown to accumulate cadmium [67]. 

Cadmium appears to have a particular affinity for endothelial cells [68,69], where it accumulates and exerts many deleterious effects. At low doses, cadmium has been shown to alter endothelial gene expression through alterations in the levels of multiple transcription factors, though the ultimate effect of these alterations is indeterminate [70,71]. At higher concentrations, cadmium has been shown to disrupt the cadherin–cadherin bonds between endothelial cells, resulting in cell contraction, endothelial disruption, and increased endothelial permeability [72]. Cadmium also seems to induce endothelial disruption through a variety of mechanisms, including p53 and DNA damage-dependent necroptosis [73], as well as p38 mitogen-activated protein kinase (MAPK38) dependent apoptosis [74,75]. Cadmium has also been shown to disrupt endothelial proliferation and angiogenesis [72,73]. It has been proposed that cadmium also exposes the endothelium to oxidative stress, though studies attempting to establish cadmium as a source of oxidative stress in this cell type have been inconsistent at best. Cadmium-induced damage to the vascular endothelium is then thought to contribute to CVD by increased permeability to immune cells and lipids, as well as the release of inflammatory cytokines, which all aid in the formation of atheromatous plaques and atherosclerosis. Cadmium has been associated with peripheral artery disease in some studies [19]. Cadmium is also associated with hypertension [72,76,77] and more atherogenic (higher low-density lipoprotein (LDL) and very-low-density lipoprotein (VLDL)) lipid profiles [78], which are known CVD risk factors. Recent animal studies have also indicated that cadmium may be prothrombotic [79]. 

A body of research is also growing that explores cadmium’s capacity for direct cardiotoxicity. Cadmium has been shown to increase oxidative stress by disruption cytochrome p450 (CYP450) and nuclear factor erythroid 2-related factor (2Nrf2) signaling in the hearts of chickens [80], decrease sarcoplasmic/endoplasmic reticulum Ca-ATPase 2 (SERCA2) expression and phosphorylated phospholamban levels resulting decreased left ventricular systolic function in male (but not female) mice [81], and cause lipid accumulation in rat cardiac myocytes, decreasing their efficiency [82,83]. Increased endoplasmic reticulum stress in cardiomyocytes has also been reported due to cadmium exposure [84], as well as cardiac ultrastructural and microstructural changes in developing and full-grown animal models [85,86]. Cadmium has also been shown to affect the cardiac conduction system via interference with the cardiac L-type calcium current, the delayed rectifier potassium current, and the sodium current in rainbow trout hearts [87]; similar effects have been observed in the L-type calcium channels of guinea pigs [88] and the delayed potassium rectifier current of cat cardiac myocytes [89].

Cadmium is a toxin with numerous, highly variegated toxic effects on the cardiovascular and many other organ systems. A characteristic pattern of low molecular weight proteinuria has been one long-standing method of detection of possible sub-clinical cadmium poisoning [90]. Tests also exist to detect cadmium in blood and urine. 

With significant toxicity affecting many major organ systems and a half-life that can last decades, cadmium accumulation and toxicity are serious threats to public health. Aggressive management to limit cadmium release and exposure in associated industries is required, as well as close monitoring of cadmium levels in food, water, and individuals in environments at high risk of contamination.

## 7. Arsenic

Arsenic is the 20th most common element in the earth’s crust, and though infamous as a poison, it is necessary in small amounts for the proper functioning of the human nervous system. Arsenic exposure occurs primarily through contaminated drinking water; this contamination can occur either naturally through processes of weathering or volcanic mobilization of natural sources of arsenic or via human activity, such as in smelting, mining, or the use of pesticides and herbicides. Cigarettes and tobacco consumption are also very common routes of arsenic exposure [91]. Arsenic poisoning via groundwater is estimated to affect more than 137 million people in more than 70 countries, with certain regions, such as the Southwestern United States and large portions of Argentina and Chile, considered “hot spots” with especially high population level exposure [55].

Arsenic is primarily absorbed via the GI tract in either a trivalent form, arsenite, or a pentavalent form, arsenate. The pentavalent form primarily enters cells through phosphate transporters, while trivalent arsenic uptake is mediated by aquaglyceroporins or sugar permeases [92]. Arsenic has been shown to cause both vascular endothelial dysfunction and direct cardiac toxicity. In endothelial cells, it mediates pro-inflammatory cytokine responses via dysregulation of tumor necrosis factor-α (TNF-α) mediated vascular cell adhesion protein 1 (VCAM-1) expression by causing arsenic-related changes in the activity of activator protein 1 (AP-1) and NF-κB [93]. Arsenic is also believed to dysregulate vascular tone by activating and upregulating nicotinamide adenine dinucleotide phosphate oxidase 2 (Nox2), reducing nitric oxide (NO) bioavailability, suppressing nitric oxide synthase (NOS) activity and expression, and promoting oxidative stress [94,95]. Additional mechanisms elucidated include induction of endothelial apoptosis through activation of epidermal growth factor (EGF), c-Jun N-terminal kinase (JNK), and MAPK38 signaling cascades, vascular injury through the neurogenic release of substance P and activation of neurokinin 1 (NK-1) receptors [95,96], and direct cytotoxicity via inactivation of protein kinase B/Akt [97]. Arsenic exposure has been shown to correlate with an increased risk of atherosclerosis in a dose-dependent fashion [98,99]. Arsenic-induced oxidative stress is thought to enhance the accumulation of oxidized lipids [100] via increased expression of Oxidized low-density lipoprotein receptor 1 (LOX-1) [101]. Arsenic exposure is also associated with hypertension [102], with proposed mechanisms including increased calcium sensitization and enhanced myosin light chain mediated vasoconstriction [79], arsenic-induced sympathetic hypersensitivity and beta-adrenoceptor stimulation [103], and enhanced expression of endothelin 1 (ET-1) messenger ribonucleic acid (mRNA) [104]. Chronic exposure to arsenic in rats and rabbits has been associated with increased peripheral vascular resistance [105].

Arsenic has also been implicated in direct cardiotoxicity, including QT prolongation and subsequent arrhythmia, ischemic heart disease, and possible apoptotic changes, though evidence for the latter remains mixed [102]. Evidence indicates that QT prolongation is potentiated by an arsenic-mediated increase in the L-type calcium current and a decrease in the inward rectifier potassium current [106]. This effect has been demonstrated in both acute [107] and chronic exposures, with chronic exposure also showing a dose-dependent relationship between arsenic poisoning and QT prolongation [108]. The association between arsenic exposure and ischemic arterial disease has been long established, with one of the first noted examples of mass arsenic poisoning presenting as the Taiwanese “blackfoot disease” caused by arsenic-induced PAD and thromboangiitis obliterans [55]; this population has also been found to have an increased incidence of ischemic heart disease [109], with a dose-response relationship also being demonstrated [110]. Studies have also implicated arsenic in ultrastructural changes [111] and developmental abnormalities in the heart [112]. Arsenic exposure has also been associated with diabetes mellitus [113] and the development of chronic kidney disease [114], both of which are known CVD risk factors.

Urine tests can be used to diagnose acute arsenic poisoning, whilst blood, hair, and nail measurements can be useful in evaluating chronic exposure [115]. Methods to remove arsenic from groundwater or provide access to arsenic-free groundwater will likely have the greatest impact in preventing arsenic-related illnesses. Several novel methods, such as the removal of arsenic from groundwater by adsorption or precipitation of biologically generated iron and manganese oxides, are currently under development. Microfiltration and the use of aquatic plants to accumulate the toxin have also been investigated [116]. Increasing awareness and limiting occupational exposures, as well as decreasing cigarette use, may also decrease arsenic exposure in the population.

## 8. Mercury

Mercury (Hg) is a ubiquitous element released into the environment through both natural and anthropogenic processes. Naturally, mercury is released via volcanic activity, geothermal activity, volatilization of oceanic mercury, and emissions from soil substrates with naturally elevated Mercury. Anthropogenic sources of environmental mercury include fossil fuel combustion, production of metals (especially gold), cement production, and waste incineration. Globally, anthropogenic mercury production is highest in Asia; natural processes tend to release mercury into the air, whilst anthropogenic processes can contaminate air, water, and soil [117]. Human exposure can occur through direct contact with sources but more commonly comes from ingesting plants or animals where prolonged mercury exposure has allowed the toxin to accumulate to higher-than-normal concentrations [118]. Aquatic exposure and accumulation of mercury in certain species of fish is an especially prominent and concerning source of mercury exposure in humans [119].

Methylmercury (MeHg) is thought to be the most toxic form of this element, as it is more readily absorbed, accumulated, and distributed throughout the body by an amino acid carrier protein after forming a methylmercury-cysteine complex [119]. The overall impact of mercury on cardiovascular health is still debated, with some studies supporting [120] or refuting [121,122] the notion that mercury is linked to CVD. Potentially harmful effects of mercury exposure may be offset by the fact that such exposure in humans is often linked to fish consumption, which has other cardiovascular benefits [119,122]. Mercury has been shown to inhibit mitochondrial function [123,124] and is thought to increase oxidative stress by inhibiting the formation of the antioxidant glutathione and increasing the production of reactive oxygen species [124,125]. This, in turn, contributes to lipid peroxidation, which may help explain the association between mercury exposure and atherosclerosis [125,126,127,128]. While some association between mercury poisoning and a prothrombotic state has been observed in animal models [129], recent research has shown no direct effect of toxic levels of mercury on coagulation in human plasma [130], indicating that prothrombotic effects may be diminished in humans or due to systemic inflammation. Mercury has been shown to decrease cardiac contractility in rat heart muscles [131] and has been associated with hypertension [132,133] and autonomic dysfunction in some populations [132,134], though this link has not been well established in other, larger studies [135]. A link between mercury and increased risk of myocardial infarction has been shown by some studies [136,137] but rejected by others [122]. No link has been established between mercury consumption and stroke, even in a large systematic review and meta-analysis that found associations between Mercury and CVD mortality and ischemic heart disease [120]. Similarly, a meta-analysis of five studies showed no distinct association between mercury and coronary artery disease [138]. Through inactivation of a necessary cofactor for catecholamine-O-methyltransferase (COMT), mercury can also inhibit the breakdown of catecholamines, leading to a clinical syndrome of catecholamine excess similar to pheochromocytoma [139,140].

Mercury is a ubiquitous toxin with wide-ranging effects both in terms of pathology and severity. Patients with hypertension and a history concerning increased exposure may be tested using specimens from hair, urine, toenails, and blood; some experts recommend obtaining all four tests to better characterize the exposure (e.g., acute vs. chronic) [137]. Improved industrial processes, better waste handling, and burning regulations and standards, and close monitoring of dietary mercury intake with increased public awareness may prove the best avenues for prevention.

## 9. Selenium

Selenium (Se) is an essential trace element that frequently contaminates sulfur-containing minerals. It is released into human environments naturally via volcanic activity and the forces of weathering. Anthropogenic sources of selenium include mining, fossil fuel refinement, and agricultural irrigation in regions of selenium-rich soil. Se is widely used in electronics, chemicals, ceramics, pharmaceuticals, and a wide variety of other industries [141].

No known cardiac toxicity to selenium has been reported [55]. Selenium deficiency may be associated with coronary artery disease, possibly due to its role as an essential trace element in many antioxidants [142]. Deficiency has also been associated with a higher mortality rate, decreased exercise tolerance, and impaired mitochondrial function in cardiac myocytes in vivo [143]. High selenium levels have also been associated with reduced risk of new-onset CHF in nonsmokers [144]. Certain selenium preparations have even been shown to mitigate cadmium-associated inflammation in the heart via the NF-kB/IkappaB kinase pathway [145]. Some studies have implicated high selenium levels in an increased incidence of metabolic syndrome and diabetes [146], but other studies find the data to be inconclusive [147]. One study has also shown some association between selenium and hypertension [148].

Selenium is most often measured in whole blood or serum. Though not known to be cardiotoxic, selenium has been shown to be toxic to livestock, aquatic wildlife, and humans in other ways. Strategies to mitigate overexposure include improved mining and agricultural runoff management techniques [141,149]. 

## 10. Zinc 

Zinc is one of the most common elements in the earth’s crust and is widely dispersed in food, water, and the air. It is commonly used to coat other metals to prevent corrosion and make dry cell batteries and is present in pennies in the United States. Zinc most commonly enters the environment through mining and refining various metals, coal burning, and the burning of waste products [150].

Zinc balance is important for heart health: deficiency of zinc has been associated with heart failure [151,152], and some evidence has shown zinc may help decrease oxidative stress and neurohormonal remodeling in the stressed myocardium [153]. Increased Zinc seems to be protective against atherosclerosis as well [154,155]. Studies have implicated both zinc excess [156] and deficiency [157] in hypertension. Zinc may also be implicated in metabolic syndrome and insulin resistance, though the exact relationship appears to be an area of ongoing research [158,159]. Maternal zinc deficiency has also been implicated in increased risk for congenital heart disease (CHD) in infants [157]. Overall, rather than a matter of toxicity, dysregulation of zinc balance seems to be the primary factor in zinc-related pathology. The body removes excess zinc by producing extra metallothionein, which binds zinc for excretion. However, metallothionein has a higher affinity for copper than zinc, and therefore, increased metallothionein production in the state of elevated zinc levels will cause relative copper deficiency [160]. This relative copper deficiency may be the actual cause of most zinc excess-related cardiovascular pathology [55].

Zinc can be detected readily in serum. Occupational safety standards, monitoring of water and food supplies for zinc excess, and enhanced recycling efforts are prominent strategies for toxicity prevention [150].

## 11. Copper

Copper (Cu) is a well-known and widely used essential trace element found in rock and mineral deposits, as well as a wide range of manufactured goods such as coins, wiring, pipes, ceramics, glaze, glass works, and electronics. It can be found in water and soil, with concentrations of copper due to human activities most prominently found near smelters, incinerators, foundries, and power plants. Copper compounds are also widely used in agriculture as fungicides, in water treatment to remove algae, and in the preservation of lumber, textiles, and tanned goods. Most toxic exposures to copper are due to anthropogenic activities such as mining, smelting, incineration of waste, or water treatment [150]. Copper exposure can also occur through medical treatments, such as prolonged total parenteral nutrition or the use of copper tubing in hemodialysis machines; certain congenital or acquired causes of hepatobiliary dysfunction, such as Wilson’s Disease, can also lead to copper accumulation and eventual toxicity [161].

Copper can exist in a cupric (2+) or Cuprous (+) state, though the former is the primary form absorbed dietarily. Once ingested, copper can bind to a number of amino acids and utilize amino acid transport proteins to disperse throughout the body. Copper functions as a cofactor for many enzymes, including cytochrome c oxidase, tyrosinase, and the important antioxidant enzyme copper–zinc–superoxide dismutase [161]. Copper excess and deficiency have both been linked to increased mortality, suggesting a U-shaped relationship between copper levels and excess morbidity/mortality [162]. Multiple observational studies have shown an association between increased levels of copper and/or the copper-binding protein ceruloplasmin and cardiovascular mortality [162,163,164], myocardial infarction, coronary artery disease, and stroke [165,166,167]. The mechanism is unclear; increases in inflammatory indices have been noted to accompany high copper levels in at least one study of patients with complex CAD [168]. Studies have associated increased copper levels with dyslipidemia, an effect thought to be secondary to the generation of free radicals by elevated copper concentrations [162,165,169]. In patients with diabetic cardiomyopathy, higher copper levels have been associated with hypertension and microvascular disease, possibly due to hyperglycemic interference with Cu-ceruloplasmin binding, which leads to increased free copper levels and greater oxidative stress through the production of free radicals [170]; at least one animal study has shown improved cardiac function in diabetic rats with cardiac dysfunction after copper chelation therapy [171]. Higher levels of copper have been reported in patients with heart failure [172,173,174], though Huang et al. found a regional variation in this phenomenon [175]. Some have speculated that ceruloplasmin may have both pro and antioxidant effects in these patients [176]. Tachycardia, as well as unifocal bigeminy and multiple ventricular extrasystoles, have been reported in patients with severe acute copper sulfate solution ingestion in at least one case report [177].

Copper levels can be assessed directly in blood/serum or via ceruloplasmin levels. As with many other metals, improved filtration and recycling techniques, more judicious use of fungicide/algaecide/pesticide chemicals, and improved techniques for the management of runoff are mainstays of prevention [150]. In addition, monitoring of copper levels in dialysis patients and patients on total parenteral nutrition (TPN), especially young infants, has proven beneficial [161].

## 12. Conclusions

**Figure 1:**
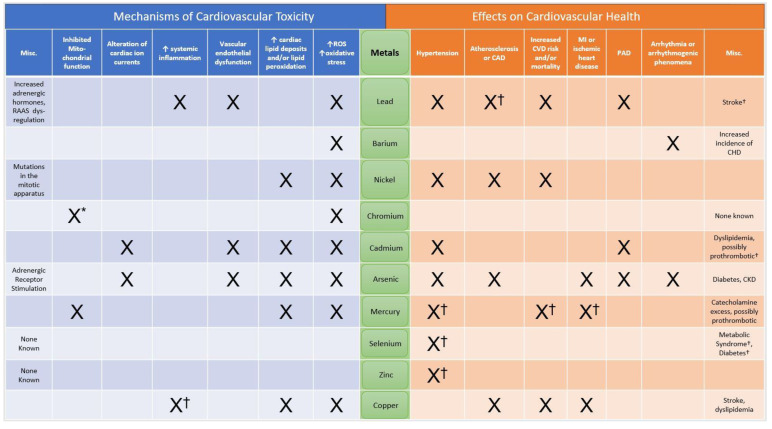
Central Image: Summary of Metal Routes of Exposure and Effects on Cardiovascular Health. ↑: “increased”; * seen at extremely high levels of exposure; † few studies or conflicting studies describing these findings.

A large body of evidence seems to support the link between exposure to certain environmental contaminant metals and worsened cardiovascular health. In this study, we sought to summarize the evidence surrounding 10 metals humans are commonly exposed to in the modern environment. Our findings are summarized in Figure 1 and detailed in Table 2. Some of these metals have broad and profound negative impacts on the cardiovascular system, while others appear to be largely benign or even essential for the proper functioning of the cardiovascular or other organ systems. We hope this review has provided a useful summary of the current evidence for clinicians and will inspire more much-needed research, especially clinical studies, to assess the rates and routes of contaminant metal exposure and the relationship between these metals and CVD. 

## Figures and Tables

**Table 1:** Metal sources and prevention strategies.

Metals	Sources	Prevention Strategies
Lead	House paint, gasoline, cosmetics and medications, lead glazed ceramics, lead utensils, lead water pipes	Routine blood lead monitoring in vulnerable communities, lead pipe and paint replacement, regulations improving lead levels requirements in gasoline, improving awareness and manufacturing processes to avoid using lead
Barium	Water contamination. Ingestion of contaminated food.	Improved studies to determine appropriate thresholds for barium toxicity
Nickel	Drinking water, food, and soil contamination. Environmental pollution in fuels, industry, and waste products.	EPA monitoring of occupational nickel and water nickel exposure. More studies to determine toxicity
Chromium	Natural trace amounts in most living creatures. Leather tanning, textile dying, paint pigmenting, wood preservation, and metal plating whose waste products cause water, soil, and food contamination. Low level airborne exposure in subway systems and the above occupations.	Improvement of occupational safety standards and improved waste disposal standards. Soil–plant barriers near dumping sites that use chromium avid plants to absorb and reduce chromium into less toxic forms.
Cadmium	Zinc and lead ores. Phosphate fertilizers. Ingested through contamination of food and water. Occupational exposure in smelting, battery, and pigment production industries. Cigarette smoke.	Smoking/tobacco use reduction. Aggressive strategies to monitor of food and water sources and prevention of use of contaminated sources. Aggressive regulation to prevent cadmium dumping in associated industries.
Arsenic	Drinking water contaminated through natural processes such as weathering or volcanic eruption, and through smelting, mining, or the use of pesticides and herbicides.	Groundwater cleansing through microfiltration, the use of plants to accumulate toxin, or processes involving adsorption of biologically generated iron and manganese oxides. Increasing awareness and limiting use of contaminated water sources. Decreased use of tobacco products.
Mercury	Natural occurrence through volcanic activity, geothermal activity, volatilization of oceanic mercury, and emissions from soil with naturally elevated mercury. Gold smelting, cement production, and waste incineration. Most human exposure through consumption of plants and animals, especially fish, in which the toxin has accumulated.	Improved industrial processes and waste handling to limit mercury contamination of the environment. Close monitoring of dietary lead levels and increased public awareness and avoidance of contaminated food sources.
Selenium	Contaminate of sulfur containing minerals released naturally through volcanic activity and weathering. Anthropogenic sources include mining, fossil fuel refinement, and agricultural irrigation.	Improved mining and agricultural runoff management.
Zinc	Widely dispersed in food, water, and air. Metal coating, battery cells, and pennies. Mining and refining, coal burning, and burning of waste products.	Occupational safety standards, monitoring of food and water supplies for excess zinc, and enhanced recycling processes.
Copper	Coins, wiring, pipes, ceramics, glaze, glass works, electronics, fungicides/herbicides, lumber preservation, textiles, tanned goods. Soil and water contamination through smelting, incinerating, powerplants, and water treatment to remove algae. Prolonged TPN, hemodialysis.	Improved filtration and recycling techniques, improved regulation of the use of fungicides and herbicide, and improved runoff management. Close monitoring of copper in certain patient’s such as those on hemodialysis or undergoing prolonged TPN use are especially useful.

**Table 2:** Select studies on contaminant metals and CVD, sorted by metal.

Lead				
Study/Authors/Year	Study Type	Study Population	Findings	Limitations
Schober SE, Mirel LB, Graubard BI, Brody DJ, Flegal KM. from ref. [17]	Cohort	9757 participants 40+ years old from the NHANES study	“Using blood lead levels < 5 µg/dL as the referent, we determined that the relative risk of mortality from all causes was 1.24 [95% confidence interval (CI), 1.05–1.48] for those with blood levels of 5–9 µg/dL and 1.59 (95% CI, 1.28–1.98) for those with blood levels ≥ 10 µg/dL (*p* for trend < 0.001). For all ages combined, the estimated relative risk of mortality from cardiovascular disease was 1.20 (95% CI, 0.93–1.55) for those with blood lead levels of 5–9 μg/dL and 1.55 (95% CI, 1.16–2.07) for those with blood lead levels of ≥10 μg/dL (test for trend, *p* < 0.01)”	Observational study, Population restricted USA, only one lead level used (not able to differentiate acute from chronic/continuous exposure), socioeconomic status (SES) confounders may not be fully accounted for (study used education and family income)
Navas-Acien A, Selvin E, Sharrett AR, Calderon-Aranda E, Silbergeld E, Guallar E. from ref. [19]	Cross Sectional	2125 participants 40+ years old in the 1999–2000 NHANES survey	“After adjustment for demographic and cardiovascular risk factors, the Odds ratios (ORs) of peripheral arterial disease comparing quartiles 2 to 4 of lead with the lowest quartile were 1.63 (95% CI, 0.51 to 5.15), 1.92 (95% CI, 0.62 to 9.47), and 2.88 (95% CI, 0.87 to 9.47), respectively (*p* for trend = 0.02). The corresponding ORs for cadmium were 1.07 (95% CI, 0.44 to 2.60), 1.30 (95% CI, 0.69 to 2.44), and 2.82 (95% CI, 1.36 to 5.85), respectively (*p* for trend = 0.01). The OR of peripheral arterial disease for current smokers compared with never smokers was 4.13. Adjustment for lead reduced this OR to 3.38, and adjustment for cadmium reduced it to 1.84.”	Observational Study, Population restricted to US, possible confounding by SES or other unmeasured pollutants, single blood measurements were used for lead and cadmium, study only able to assess prevalent, not incident cases of PAD
Pocock SJ, Shaper AG, Ashby D, Delves HT, Clayton BE. from ref. [20]	Cross Sectional + Cohort	7371 men aged 40–59 from 24 British towns	“Cross-sectional data indicate that an estimated mean increase of 1.45 mm Hg in systolic blood pressure occurs for every doubling of blood lead concentration with a 95% confidence interval of 0.47 to 2.43 mm Hg. After 6 years of follow-up, 316 of these men had major ischemic heart disease, and 66 had a stroke. After allowance for the confounding effects of cigarette smoking and town of residence there is no evidence that blood lead is a risk factor for these cardiovascular events.”	Observational study, population restricted to British men, not all confounders possibly accounted for.
Cheng Y, Schwartz J, Vokonas PS, Weiss ST, Aro A, Hu H. from ref. [23]	Cross Sectional	775 men who participated in the Normative Aging Study (age 48–93)	“Bone lead levels were found to be positively associated with heart rate– corrected QT and QRS intervals, especially in younger men. Specifically, in men < 65 years of age, a 10 μg/g increase in tibia lead was associated with an increase in the QT interval of 5.03 ms (95% confidence interval [CI], 0.83 to 9.22) and with an increase in the QRS interval of 4.83 ms (95% CI, 1.83 to 7.83) in multivariate regression models. In addition, an elevated bone lead level was found to be positively associated with an increased risk of intraventricular block in men < 65 years of age and with an increased risk of atrioventricular (AV) block in men ≥ 65 years of age. After adjustment for age and for serum high-density lipoprotein (HDL) level, a 10 μg/g increase in tibia lead was associated with an odds ratio (OR) of 2.23 (95% CI, 1.28 to 3.90) for intraventricular block in men < 65 years of age and with an OR of 1.22 (95% CI, 1.02 to 1.47) for AV block in men ≥ 65 years of age. Blood lead level was not associated with any of the electrocardiogram (ECG) outcomes examined.”	Observational Study, population restricted to men from the Boston area (USA), only 1 cumulative bone-lead measurement taken
Navas-Acien A, Guallar E, Silbergeld EK, Rothenberg SJ. from ref. [24]	Systematic Review	12 studies of lead and CVD in general populations, 18 studies of lead and CVD mortality in occupational populations, and 5 studies of lead and intermediate cardiovascular outcomes	“A positive association of lead exposure with blood pressure has been identified in numerous studies in different settings, including prospective studies and in relatively homogeneous socioeconomic status groups. Several studies have identified a dose-response relationship. Although the magnitude of this association is modest, it may be underestimated by measurement error. The hypertensive effects of lead have been confirmed in experimental models. Beyond hypertension, studies in general populations have identified a positive association of lead exposure with clinical cardiovascular outcomes (cardiovascular, coronary heart disease, and stroke mortality; and peripheral arterial disease), but the number of studies is small. In some studies these associations were observed at blood lead levels < 5 µg/dL.”	Largely based on observational studies, quantitative analysis limited
**Barium**				
**Study/Authors/Year**	**Study Type**	**Study population**	**Findings**	**Limitations**
Wones RG, Stadler BL, Frohman LA. from ref. [28]	Experimental Trial	11 men	“There were no changes in morning or evening systolic or diastolic blood pressures, plasma cholesterol or lipoprotein or apolipoprotein levels, serum potassium or glucose levels, or urine catecholamine levels. There were no arrythmias related to barium exposure detected on continuous electrocardiographic monitoring. A trend was seen toward increased total serum calcium levels with exposure to barium, which was of borderline statistical significance and of doubtful clinical significance. In summary, drinking water barium at levels of 5 and 10 ppm did not appear to affect any of the known modifiable cardiovascular risk factors.”	Extremely small sample size, no control group, barium-only study at very dilute exposure concentrations over very short period of time, subjects allowed to drink infinite distilled water after completing daily dose of barium-laced water
Perry HM Jr, Kopp SJ, Perry EF, Erlanger MW. from ref. [29]	Experimental animal study	195 Long–Evans rats	“Average systolic pressure increased significantly after exposure to 100 ppm barium for 1 mo or longer and after exposure to 10 ppm barium for 8 mo or longer. After 4 or 76 mo, barium exposure failed to alter organ weights or tissue concentrations of calcium, magnesium, sodium, or potassium; however, both 10 and 100 ppm barium resulted in significant increases in tissue barium. Rats exposed to 100 ppm Ba for 16 mo exhibited depressed rates of cardiac contraction and depressed electrical excitability in the heart. Hearts from these maximally exposed rats also had significantly lower ATP content and phosphorylation potential, as measured by 31 P NMR spectros-copy.”	Animal study, relatively small sample size
Brenniman GR, Namekata T, Kojola WH, Carnow BW, Levy PS. from ref. [30]	Retrospective cohort	Communities in Illinois between 1971–1975	“Results of this mortality study revealed that the high barium communities had significantly higher (*p* < 0.05) death rates for “all cardiovascular diseases” and “heart disease” compared to the low barium communities.	Observational study, population restricted to Illinois cities, many possible confounders that were not controlled for (e.g., differentials in population change, water softener use, etc.)
**Nickel**				
**Study/Authors/Year**	**Study Type**	**Study population**	**Findings**	**Limitations**
Zhang N, Chen M, Li J, Deng Y, Li SL, Guo YX, Li N, Lin Y, Yu P, Liu Z, Zhu J. from ref. [35]	Case-Control	399 cases (pregnant women with fetal congenital heart disease (CHD)) and 490 controls (pregnant females without fetal CHD)	The median concentrations of nickel were 0.629 ng/mg, *p* < 0.05 (adjusted odds ratio [aOR], 1.326; 95% CI, 1.003–1.757) and 0.178 ng/mg, *p* < 0.05 (aOR, 2.204; 95% CI, 0.783–6.206), in maternal hair and in fetal placental tissue in the CHD group, respectively. Significant differences in the level of nickel in hair were also found in the different CHD subtypes including septal defects (*p* < 0.05), conotruncal defects (*p* < 0.05), right ventricular outflow tract obstruction (*p* < 0.01), and left ventricular outflow tract obstruction (*p* < 0.05). Dramatically different nickel concentrations in fetal placenta tissue were found in cases with other heart defects (*p* < 0.05).”	Observational study, Restricted to Chinese women, unknown how well placental or maternal hair nickel levels actually correspond to fetal exposure, possible confounding with toxic effects from other metals
Cheek, J., Fox, S.S., Lehmler, HJ. et al. from ref. [39]	Cross Sectional	2739 adults 18+ years old from the NHANES 2017–20	“Urinary nickel concentrations were higher in individuals with CVD (weighted median 1.34 μg/L) compared to those without CVD (1.08 μg/L). After adjustment for demographic, socioeconomic, lifestyle, and other risk factors for CVD, the ORs (95% CIs) for CVD compared with the lowest quartile of urinary nickel were 3.57 (1.73–7.36) for the second quartile, 3.61 (1.83–7.13) for the third quartile, and 2.40 (1.03–5.59) for the fourth quartile. Cubic spline regression revealed a non-monotonic, inverse U-shaped, association between urinary nickel and CVD (P_nonlinearity_ < 0.001).”	Observational study, population restricted to US adults, only used one spot urinary nickel sample
Lind, P. M., Olsén, L., and Lind, L. from ref. [40]	Cross Sectional	1016 adults aged 70 in the Prospective Investigation of the Vasculature in Uppsala Seniors (PIVUS) study	“Nickel levels were related to the number of carotid arteries with plaques in an inverted U-shaped manner after multiple adjustment for gender, waist circumference, body mass index, fasting blood glucose, systolic and diastolic blood pressure, HDL and LDL cholesterol, serum triglycerides, smoking, antihypertensive treatment and statin use (*p* = 0.026).”	Observational study, population limited to Caucasians from the PIVUS study aged 70, study had a moderate participation rate that may have led to some mild bias
Shi, P., Liu, S., Xia, X., Qian, J., Jing, H., Yuan, J., Zhao, H., Wang, F., Wang, Y., Wang, X., Wang, X., He, M., and Xi, S. from ref. [41]	Cross Sectional	865 participants from 6 factories in northeastern China	“A Bayesian Kernel Machine Regression indicated a hormetic triphasic dose-response relationship between eight urinary metals (Co, Cr, Ni, Cd, As, Fe, Zn, and Pb) and hypertension.”	Observational Study, population restricted to male factory workers in one region of northeastern China, only one urinary metal measurement was performed, hypertension status based on self-report and single laboratory measurement, antihypertensive medication use not considered
**Chromium**				
**Study/Authors/Year**	**Study Type**	**Study population**	**Findings**	**Limitations**
Bai, J., Xun, P., Morris, S., Jacobs, J., Liu, K., and He, K. from ref. [48]	Retrospective Cohort	3648 adults from CARDIA study, age 20–32 years	“multivariable-adjusted hazard ratio (HR) (95% confidence interval [CI]) of metabolic syndrome comparing the highest to the lowest quartiles of toenail chromium levels was 0.80 (0.66–0.98; Plinear trend = 0.006). The adjusted HRs were 0.82 (0.68–0.98; Ptrend = 0.045) for having abnormal triglycerides levels and 0.75 (0.64–0.88; Ptrend = 0.030) for having abnormal HDL cholesterol levels.”	Observational study, chromium only measured once at beginning of study, population recruited from urban areas of the USA
Chen, J., Kan, M., Ratnasekera, P., Deol, L. K., Thakkar, V., and Davison, K. from ref. [49]	Cross Sectional	2982 adults 40+ years of age from 2016 NHES survey	“The odds of CVDs were 1.89 times higher for males who had blood chromium levels < 0.7 μg/L (aOR = 1.89, 95% CI: 1.24–2.90) compared to those who had levels within the normal range. Males who had low blood chromium levels (aOR = 0.40, 95% CI: 0.21–0.74) had lower odds of depression compared to those with normal blood chromium levels. The odds of diabetes mellitus (DM) was 2.58 times higher for males and 1.99 times higher for females with low blood chromium levels compared to those with normal blood chromium levels (aOR = 2.58 (male), 1.99 (female), *p*’s < 0.001).”	Observational study, no longitudinal data, population restricted to noninstitutionalized civilian resident population of USA
Gutiérrez-Bedmar, M., Martínez-González, M. Á., Muñoz-Bravo, C., Ruiz-Canela, M., Mariscal, A., Salas-Salvadó, J., Estruch, R., Corella, D., Arós, F., Fito, M., Lapetra, J., Serra-Majem, L., Pintó, X., Alonso-Gómez, Á., Portoles, O., Fiol, M., Bulló, M., Castañer, O., Ros, E., and Gómez-Gracia, E. from ref. [50]	Nested Case-Control	147 Cases with CVD, 271 Controls	“The fully adjusted OR for the highest vs. lowest quartile of toenail Cr was 0.54 (95% CI: 0.26–1.14; Ptrend = 0.189) for the nested case-control study. On stratification for diabetes mellitus (DM), OR was 1.37 (95% CI: 0.54–3.46; Ptrend = 0.364) for the DM group, and 0.25 (95% CI: 0.08–0.80; Ptrend = 0.030) for the non-DM group (*p* for interaction = 0.078).”	Observational Study, chromium only measured once at beginning of study, population restricted to Spanish Adults aged 55–80, sample size relatively small
Guallar, E., Jiménez, F. J., van ’t Veer, P., Bode, P., Riemersma, R. A., Gómez-Aracena, J., Kark, J. D., Arab, L., Kok, F. J., and Martín-Moreno, J. M. from ref. [52]	Case-Control Study	684 male cases with first time MI, 724 male controls	“Average toenail chromium concentrations were 1.10 μg/g in cases (95% confidence interval: 1.01, 1.18) and 1.30 μg/g in controls (95% CI: 1.21, 1.40). Multivariate odds ratios for quintiles 2–5 were 0.82 (95% CI: 0.52, 1.31), 0.68 (95% CI: 0.43, 1.08), 0.60 (95% CI: 0.37, 0.97), and 0.59 (95% CI: 0.37, 0.95).”	Observational Study, Males studied only, chromium measured only once at the start of the study, population restricted to men form 8 European countries and Israel, only studied nonfatal MI
ROEBACK, J. R., HLA, K. M., CHAMBLESS, L. E., and FLETCHER, R. H. from ref. [53]	Randomized Control Trial	72 men receiving beta blockers (63, 88%, completed the study)	“After 8-week treatment phase of total daily chromium dose of 600 micrograms in 3 daily doses, mean baseline levels of HDL and total cholesterol (±SD) were 0.93 ± 0.28 mmol/L and 6.0 ± 1.0 mmol/L (36 ± 11.1 mg/dL and 232 ± 38.5 mg/dL), respectively. The difference between groups in adjusted mean change in HDL cholesterol levels, accounting for baseline HDL cholesterol levels, age, weight change, and baseline total cholesterol levels, was 0.15 mmol/L (5.8 mg/dL) (*p* = 0.01) with a 95% Cl showing that the treatment effect was greater than +0.04 mmol/L (+1.4 mg/dL). Mean total cholesterol, triglycerides and body weight did not change significantly during treatment for either group. Compliance as measured by pill count was 85%, and few side effects were reported. Two months after the end of treatment, the between-group difference in adjusted mean change from baseline to end of post-treatment follow-up was −0.003 mmol/L (−0.1 mg/dL).”	Restricted to males on beta blockers at a single VA medical center (Durham VAMC). Some significant differences in treatment vs. placebo group (e.g., 49% of placebo reported alcohol use compared to just 29% in treatment group; 26% of treatment group was post-MI vs. just 11% of placebo group)
Price Evans, D. A., Tariq, M., Dafterdar, R., Al Hussaini, H., and Sobki, S. H. from ref. [54]	Randomized control trial–Animal Study	20 male adult New Zealand white Rabbits (4 normal diet, 8 high cholesterol diet w/IM NaCl, 8 with high cholesterol diet w/IM CrCl3)	“The size of lipid deposits in the coronary vasculature of the hypercholesterolemic rabbits were greatly reduced after intramuscular chromium chloride injections. Lipid deposits in the ascending aorta were similarly reduced, as well as the serum cholesterol concentrations. The terminal serum chromium concentrations in the chromium-treated group were in the range of 3258–4513 μg/L, whereas, in the untreated animals, the concentrations were 3.2 to 6.3 μg/L. The general condition of the chromium-treated animals was good and they were continuing to gain weight up to the time they were killed. Liver function tests had become abnormal even though there was no evidence of hepatic histopathological lesions specifically affecting the chromium-treated group. The kidney function tests and histopathology were normal.”	Animal study, very small sample size
**Cadmium**				
**Study/Authors/Year**	**Study Type**	**Study population**	**Findings**	**Limitations**
Menke, A., Muntner, P., Silbergeld, E. K., Platz, E. A., and Guallar, E. from ref. [64]	Retrospective Cohort	13,958 NHANES III participants	“After multivariable adjustment, the hazard ratios [95% confidence interval (CI)] for all-cause, cancer, cardiovascular disease, and coronary heart disease mortality associated with a 2-fold higher creatinine-corrected urinary cadmium were, respectively, 1.28 (95% CI, 1.15–1.43), 1.55 (95% CI, 1.21–1.98), 1.21 (95% CI, 1.07–1.36), and 1.36 (95% CI, 1.11–1.66) for men and 1.06 (95% CI, 0.96–1.16), 1.07 (95% CI, 0.85–1.35), 0.93 (95% CI, 0.84–1.04), and 0.82 (95% CI, 0.76–0.89) for women.”	Observational study, Population restricted to civilian noninstitutionalized adults in the USA, use of single spot urine cadmium samples, possible confounding by smoking status (though adjusted for smoking status and pack years and results were similar for the subgroup analysis of never-smoking men)
Messner, B., Knoflach, M., Seubert, A., Ritsch, A., Pfaller, K., Henderson, B., Shen, Y. H., Zeller, I., Willeit, J., Laufer, G., Wick, G., Kiechl, S., and Bernhard, D. from ref. [74]	Human Cross Sectional and Animal Randomized Control Trial	195 healthy women age 18–22 in the Atherosclerosis Risk Factors in Female Youngsters (ARFY) study; Female ApoE KO mice (number not stated)	“In the young women, cadmium (Cd) level was independently associated with early atherosclerotic vessel wall thickening (intima-media thickness exceeding the 90th percentile of the distribution; multivariable OR 1.6 [1.1.–2.3], *p* = 0.016). In line, Cd-fed Apolipoprotein E knockout mice yielded a significantly increased aortic plaque surface compared to controls (9.5 versus 26.0 mm, *p* < 0.004).”	Smaller sample size, human part of study observational, only females tested, clinical significance of findings uncertain as patient outcomes were not charted over time
Eum, K.-D., Lee, M.-S., and Paek, D. from ref. [78]	Cross Sectional	958 men and 944 women in the 2005 Korean National Health and Nutrition Examination Survey (KNHANES)	“After adjusting for covariates, the odds ratio of hypertension comparing the highest to the lowest tertile of cadmium in blood was 1.51 (95% confidence interval 1.13 to 2.05), and a dose–response relationship was observed. Systolic, diastolic, and mean arterial blood pressure were all positively associated with blood cadmium level, and this effect of cadmium on blood pressure was markedly stronger when the kidney function was reduced.”	Observational Study, Population restricted to Korean adults, only a single measurement of cadmium was taken at one point in time.
Maria Tellez-Plaza, Navas-Acien, A., Crainiceanu, C. M., and Guallar, E. from ref. [79]	Cross Sectional	US adults NHANES participants with blood (n = 10,991) and urine (n = 3496) cadmium levels recorded	“After multivariable adjustment, the average differences in systolic and diastolic blood pressure comparing participants in the 90th vs. 10th percentile of the blood cadmium distribution were 1.36 mmHg [95% confidence interval (CI), −0.28 to 3.00] and 1.68 mmHg (95% CI, 0.57–2.78), respectively. The corresponding differences were 2.35 mmHg and 3.27 mmHg among never smokers, 1.69 mmHg and 1.55 mmHg among former smokers, and 0.02 mmHg and 0.69 mmHg among current smokers. No association was observed for urine cadmium with blood pressure levels, or for blood and urine cadmium with the prevalence of hypertension.”	Observational study, restricted to US adults >20 years, only single blood and urine measurements of cadmium were made, most participants had blood cadmium levels close to the limit of detection
**Arsenic**				
**Study/Authors/Year**	**Study Type**	**Study population**	**Findings**	**Limitations**
Hsieh, Y.-C., Hsieh, F.-I., Lien, L.-M., Chou, Y.-L., Chiou, H.-Y., and Chen, C.-J. from ref. [98]	Case-Control	479 residents (235 cases, 244 controls) from Noretheastern Taiwan	“Significant crude and age-and-gender adjusted odds ratios [CI] for Atherosclerosis in those exposed to elevated concentrations of Arsenic > 50 μg/L in well water (crude: 2.0 [1.1–3.8] Ptrend = 0.0054, adjusted 2.4 [1.2–4.6] Ptrend = 0.0049) and for those with cumulative arsenic exposure > 1.1 mg/L/year (crude: 1.6 [1.0–26], adjusted 1.9 [1.1–3.1]).”	Observational study, family history of stroke and CVD not included, used arsenic in well water instead of direct urinary/serum arsenic measurements, population restricted to Taiwanese citizens, did not norm exposures by body weight
Wang, Y.-H., Wu, M.-M., Hong, C.-T., Lien, L.-M., Hsieh, Y.-C., Tseng, H.-P., Chang, S.-F., Su, C.-L., Chiou, H.-Y., and Chen, C.-J. from ref. [99]	Case-Control	605 resident (289 men, 316 women) from Northeastern Taiwan	“A significant age-and gender-adjusted odds ratio of 3.3 for the development of carotid atherosclerosis was observed among the high-arsenic exposure group who drank well water containing arsenic at levels > 50 μg/L.”	Observational Study, population restricted to Taiwanese citizens, info on familial history of stroke, CVD, and LDL were not collected, used arsenic in well water instead of direct urinary/serum arsenic measurements
Mumford, J. L., Kegong Wu, Xia, Y., Richard Kwok, Zhihui Yang, Foster, J., and William E. Sanders Jr. from ref. [108]	Cross Sectional	313 residents from Ba Men, Inner Mongolia	“The prevalence rates of QT prolongation and water arsenic concentrations showed a dose-dependent relationship (*p* = 0.001). The prevalence rates of QTc prolongation were 3.9, 11.1, 20.6% for low, medium, and high arsenic exposure, respectively. QTc prolongation was also associated with sex (*p* < 0.0001) but not age (*p* = 0.486) or smoking (*p* = 0.1018). Females were more susceptible to QT prolongation than males.”	Observational study, Population restricted to Inner Mongolia, relatively small sample size, analysis for some confounding factors, such as medication use, not performed
Chen, C.-J., Chiou, H.-Y., Chiang, M.-H., Lin, L.-J., and Tai, T.-Y. from ref. [110]	Cohort	Residents in 60 villages of the area of Taiwan with Endemic Arseniasis (1,355,915 person-years)	“Based on 1 355 915 person-years and 217 ischemic heart disease (IHD) deaths, the cumulative IHD mortalities from birth to age 79 years were 3.4%, 3.5%, 4.7%, and 6.6%, respectively, for residents who lived in villages in which the median arsenic concentrations in drinking water were <0.1, 0.1 to 0.34, 0.35 to 0.59, and greater or equal to 0.6 mg/L. A cohort of 263 patients affected with blackfoot disease (BFD), a unique arsenic-related peripheral vascular disease, and 2293 non-BFD residents in the endemic area of arseniasis were recruited and followed up for an average period of 5.0 years. There was a monotonous biological gradient relationship between cumulative arsenic exposure through drinking artesian well water and IHD mortality. The relative risks were 2.5, 4.0, and 6.5, respectively, for those who had a cumulative arsenic exposure of 0.1 to 9.9, 10.0 to 19.9, and greater or equal to 20.0 mg/L-years compared with those without the arsenic exposure after adjustment for age, sex, cigarette smoking, body mass index, serum cholesterol and triglyceride levels, and disease status for hypertension and diabetes through proportional-hazards regression analysis. BFD patients were found to have a significantly higher ISHD mortality than non-BFD residents, showing a multivariate-adjusted relative risk of 2.5 (95% CI, 1.1 to 5.4).”	Obserational Study, Restricted to Taiwanese populations in area with endemic high exposure (e.g., areas with “blackfoot disease”)
Zheng, L. Y., Umans, J. G., Yeh, F., Francesconi, K. A., Goessler, W., Silbergeld, E. K., Bandeen-Roche, K., Guallar, E., Howard, B. V., Weaver, V. M., and Navas-Aciena, A. from ref. [114]		American Indian adults age 45–74 in the Strong Heart Study (3851 in cross sectional analysis, 3119 in rospective analysis)	“The adjusted odds ratio (OR; 95% confidence interval) of prevalent CKD for an interquartile range in total arsenic was 0.7 (0.6, 0.8), mostly due to an inverse association with inorganic arsenic (OR 0.4 [0.3, 0.4]). Monomethylarsonate and dimethylarsinate were positively associated with prevalent CKD after adjustment for inorganic arsenic (OR 3.8 and 1.8). The adjusted hazard ratio of incident CKD for an IQR in sum of inorganic and methylated arsenic was 1.2 (1.03, 1.41). The corresponding HRs for inorganic arsenic, monomethylarsonate, and dimethylarsinate were 1.0 (0.9, 1.2), 1.2 (1.00, 1.3), and 1.2 (1.0, 1.4).”	Observational study, Population restricted to mostly rural American Indians, eGFR limited to 3 measurements during study, population has high prevalence of comorbidities (including CKD, obesity, and diabetes)
**Mercury**				
**Study/Authors/Year**	**Study Type**	**Study population**	**Findings**	**Limitations**
Hu, X. F., Lowe, M., and Chan, H. M. from ref. [120]	Meta-Analysis and Systematic Review	14 studies with more than 34,000 participants in 17 countries	“Hg exposure was associated with an increase in nonfatal ischemic heart disease (relative risk (RR): 1.21 (0.98, 1.50)), all-cause mortality (RR: 1.21 (0.90, 1.62)), CVD mortality (RR: 1.68 (1.15, 2.45)), and mortality due to other heart diseases (RR: 1.50 (1.07, 2.11)). No association was observed between Hg exposure and stroke. A heterogeneous relationship was found between studies reporting fatal and nonfatal outcomes and between cohort and non-cohort studies. A J-shaped relationship between Hg exposure and different fatal/nonfatal outcomes was observed, with turning points at hair Hg concentrations of 1 μg/g for IHD and 2 μg/g for stroke and all CVD.”	Study populations mainly Caucasian, combined cohort and non-cohort studies when comparing nonfatal CVD events, did not include protective effects of certain nutrients (e.g., omega-3 fatty acids) that were part of the analysis in some studies
Downer, M. K., Martínez-González, M. A., Gea, A., Stampfer, M., Warnberg, J., Ruiz-Canela, M., Salas-Salvadó, J., Corella, D., Ros, E., Fitó, M., Estruch, R., Arós, F., Fiol, M., Lapetra, J., Serra-Majem, L., Bullo, M., Sorli, J. V., Muñoz, M. A., García-Rodriguez, A., … Gómez-Gracia, E. from ref. [121]	Nested Case-Control	7477 participants in the PREDIMED trial at high risk for CVD at baseline	“Mean (±SD) toenail mercury concentrations (μg per gram) did not significantly differ between cases (0.63 (±0.53)) and controls (0.67 (±0.49)). Mercury concentration was not associated with cardiovascular disease in any analysis, and neither was fish consumption or n-3 fatty acids. The fully-adjusted relative risks for the highest versus lowest quartile of mercury concentration were 0.71 (95% Confidence Interval [CI], 0.34, 1.14; *p* = 0.37) for the nested case-control study, 0.74 (95% CI, 0.32, 1.76; *p* = 0.43) within the Mediterranean diet intervention group, and 0.50 (95% CI, 0.13, 1.96; *p* = 0.41) within the control arm of the trial.”	Observational study, only used one measure of toenail mercury, possible negative confounding by association of omega-3 fatty acid with fish take, population restricted to adults enrolled in PREDIMED trial in Spain
Mozaffarian, D., Shi, P., Morris, J. S., Spiegelman, D., Grandjean, P., Siscovick, D. S., Willett, W. C., and Rimm, E. B. from ref. [122]	Prospective cohort	3427 participants with matched risk-set-sample controls according to age, sex, race, and smoking status	“0.23 μg per gram (interdecile range, 0.06 to 0.94) in the case participants and 0.25 μg per gram (interdecile range, 0.07 to 0.97) in the controls. In multivariate analyses, participants with higher mercury exposures did not have a higher risk of cardiovascular disease. For comparisons of the fifth quintile of mercury exposure with the first quintile, the relative risks were as follows: coronary heart disease, 0.85 (95% confidence interval [CI], 0.69 to 1.04; *p* = 0.10 for trend); stroke, 0.84 (95% CI, 0.62 to 1.14; *p* = 0.27 for trend); and total cardiovascular disease, 0.85 (95% CI, 0.72 to 1.01; *p* = 0.06 for trend). Findings were similar in analyses of participants with low selenium concentrations or low overall fish consumption and in several additional sensitivity analyses.”	Observational Study, population restricted to United states, only used one measure of toenail mercury, possible negative confounding by association of omega-3 fatty acid with fish take
Valera, B., Dewailly, E., and Poirier, P. from ref. [132]	Cross Sectional	280 Nunavik Adults age 40+	“Mercury was negatively correlated with low frequency LF (r = −0.18; *p* = 0.02), the standard deviation of RR intervals (SDNN) (r = −0.14; *p* = 0.047) and the coefficient of variation in RR intervals (CVRR) (r = −0.18; *p* = 0.011) while correlations with other HRV parameters did not reach statistical significance. After adjusting for confounders, the association with LF (beta = −0.006; *p* = 0.93) became non-significant. However, the association with SDNN became statistically significant (beta = −0.086; *p* = 0.026) and CVRR tended to decrease with blood mercury concentrations (beta = −0.057; *p* = 0.056). Mercury was positively correlated with SBP (r = 0.25; *p* < 0.0001) and PP (r = 0.33; *p* < 0.0001). After adjusting for confounders, these associations remained statistically significant (beta SBP = 4.77; *p* = 0.01 and beta PP = 3.40; *p* = 0.0036).”	Observational study, small sample, population restricted to Nunavik adults, low participation rate for ambulatory EKGs in the study population (possible selection bias)
Park, S. K., Lee, S., Basu, N., and Franzblau, A. from ref. [135]	Cross Sectional	2201 adults from NHANES 2003–2006 study population with urinary mercury reading	“The weighted prevalence of hypertension was 32.2%. The geometric means (95% confidence intervals) of blood total and urinary mercury were 1.03 (0.95, 1.11) μg/L and 0.51 (0.47, 0.54) μg/L, respectively. The adjusted odds ratios for a doubling increase in blood mercury and urinary mercury were 0.94 (0.87 to 1.01) and 0.87 (0.78 to 0.99), respectively, after adjusting for potential confounders.”	Observational Study, population restricted to US adults, large percentage of participants had mercury levels below the lower limit of normal (ranging for 7.4% to 25.4% depending on the year and sample type)
Mozaffarian, D., and Rimm, E. B. from ref. [138]	Meta-Analysis	1601 combined events from 5 different studies; 1 retrospective (684 events) and 4 prospective	“The overall pooled relative risk (dotted line) and 95% CI (diamond), estimated using inverse-variance random-effects meta-analysis, was 1.12 (95% CI, 0.71–1.75; *p* = 0.62), with significant heterogeneity between studies (*p* = 0.008).”	Studies extremely heterogeneous, Study populations skewed towards US and Europe, one large US study comprised mostly dentists whose occupational mercury exposure is to inorganic mercury, which may be less toxic than the methylmercury found in fish
**Selenium**				
**Study/Authors/Year**	**Study Type**	**Study population**	**Findings**	**Limitations**
Flores-Mateo, G., Navas-Acien, A., Pastor-Barriuso, R., and Guallar, E. from ref. [142]	Meta-Analysis	4 cohort studies, 11 case-control studies, and 6 clinical trials	“The pooled relative risk in a comparison of the highest with the lowest selenium concentration categories was 0.85 (95% CI: 0.74, 0.99) in cohort studies and 0.43 (0.29, 0.66) in case-control studies. In observational studies, a 50% increase in selenium concentrations was associated with a 24% (7%, 38%) reduction in coronary heart disease risk. In randomized trials, the pooled relative risk in a comparison of supplements containing selenium with placebo was 0.89 (0.68, 1.17).”	Observational studies and trials not in agreement (though tendency of clinical trials is towards agreement with observational studies). How to compare different selenium iomakers used in different studies (e.g., nail vs. blood samples) is uncertain
Bomer, N., Grote Beverborg, N., Hoes, M. F., Streng, K. W., Vermeer, M., Dokter, M. M., IJmker, J., Anker, S. D., Cleland, J. G. F., Hillege, H. L., Lang, C. C., Ng, L. L., Samani, N. J., Tromp, J., van Veldhuisen, D. J., Touw, D. J., Voors, A. A., and van der Meer, P. from ref. [143]	Retrospective Cohort	2516 patients with heart failure from the BIOSTAT-CHF study (from 69 centers in 11 European countries)	“Serum selenium concentration (deficiency) was <70 μg/L in 485 (20.4%) patients, who were older, more often women, had worse New York Heart Association class, more severe signs and symptoms of heart failure and poorer exercise capacity (6-min walking test) and quality of life (Kansas City Cardiomyopathy Questionnaire). Selenium deficiency was associated with higher rates of the primary endpoint [hazard ratio (HR) 1.23; 95% confidence interval (CI) 1.06–1.42] and all-cause mortality (HR 1.52; 95% CI 1.26–1.86).”	Observational studies, Population restricted to Europeans in BIOSTAT-CHF, reference ranges for selenium had some variation between countries (author discretion was used to define deficiency at <70 μg/L)
Al-Mubarak, A. A., Grote Beverborg, N., Suthahar, N., Gansevoort, R. T., Bakker, S. J. L., Touw, D. J., de Boer, R. A., van der Meer, P., and Bomer, N. from ref. [144]	Retrospective cohort	5973 participants in the Dutch PREVEND study	“Serum selenium was measured in 5973 subjects and mean selenium concentration was 84.6 (±19.5) μg/L. Mean age was 53.6 (±12.1) years and 3103 subjects (52%) were females. Median follow-up period was 8.4 years. Selenium levels associated positively with female sex, higher total cholesterol and glucose concentrations, and associated negatively with incidence of anemia, iron deficiency, current smoking, increasing C-reactive protein levels, and higher body mass index. Univariate analysis on all subjects showed no association of continuous selenium concentrations, per 10 μg/L increase, with the composite endpoint (Hazard Ratio [HR] = 0.96, 95% Confidence interval [CI]: 0.87 to 1.06, *p* = 0.407). However, significant interaction with smoking status was observed. In non-smoking subjects (N = 4288), continuous selenium concentrations were independently associated with reduced mortality risk (HR = 0.87, 95% CI: 0.79 to 0.96, *p* = 0.005), lower risk of new-onset HF (HR = 0.82, 95% CI: 0.69 to 0.96, *p* = 0.017), as well as reduced risk of the composite endpoint (HR = 0.86, 95% CI: 0.79 to 0.94, *p* = 0.001). In smoking subjects, no associations were found.”	Observational Study, Population restricted to Dutch patients, relatively small number of patients with optimal selenium levels (>123 μg/L).
Lu, C.-W., Chang, H.-H., Yang, K.-C., Chiang, C.-H., Yao, C.-A., and Huang, K.-C. from ref. [146]	Case-Control	1165 40+ Adults at National Taiwan University Hospital	“The mean serum selenium concentration was 96.34 ± 25.90 μg/L, and it was positively correlated with waist circumference, systolic blood pressure, triglycerides, fasting glucose, and homeostatic model assessment insulin resistance (HOMA-IR) in women, but it was only correlated with fasting glucose and HOMA-IR in men. After adjustment, the odds ratios (ORs) of having metabolic syndrome increased with the selenium quartile groups (for trend: <0.05), especially in women.”	Observational study, dietary data not recorded, population restricted to National Taiwan University Hospital ambulatory patients
Retondario, A., Fernandes, R., Rockenbach, G., Alves, M. de A., Bricarello, L. P., Trindade, E. B. S. de M., and Vasconcelos, F. de A. G. de. from ref. [147]	Systematic Review	6 studies (1 double blind RCT, 5 Cross Sectional)	“Three studies found no association between Selenium intake and metabolic syndrome; two of them found an inverse association; and one study found a direct association between Selenium intake and metabolic syndrome. One study also showed an inverse association between Selenium intake and the prevalence of high waist circumference, high diastolic blood pressure, and hyperglycemia in women.”	Not Quantitative. Relatively small number of studies considered
Bastola, M. M., Locatis, C., Maisiak, R., and Fontelo, P. from ref. [148]	Cross Sectional	6683 participants (3289 males and 3394 females) in the NHANES 2011–2016 database	“Findings showed a significant positive association between serum selenium levels and hypertension but not serum zinc and copper. At optimal levels for transport and distribution, serum selenium levels of 120 mu g/L or higher (reference level 70–150 mu g/L) were significantly associated with hypertension (OR = 1.46, 95% CI = 1.29–1.66) after adjusting for confounding factors. At serum selenium level greater than 150 mu g/L, the association with hypertension strengthened (OR = 1.69, 95% CI = 1.32–2.17).”	Observational study, population restricted to the USA participants, patients were defined as hypertensive based soley on the average of 3 blood pressure readings, regardless of antihypertensive drug intake
**Zinc**				
**Study/Authors/Year**	**Study Type**	**Study population**	**Findings**	**Limitations**
Rosenblum, H., Wessler, J. D., Gupta, A., Maurer, M. S., and Bikdeli, B. from ref. [151]	Systematic Review	33 studies pertaining to zinc deficiency and heart failure (25 original articles, 8 review articles)	“Systematic review of the literature is suggestive of pathobiological pathways linking zinc deficiency to the development and progression of heart failure (HF) syndromes. Some available studies are suggestive of the potential therapeutic role of zinc supplementation, which warrants further confirmation in subsequent prospective (ideally randomized) trials.”	Nearly all observational studies, no quantitative analysis, majority of identified studies were small in size.
Yu, X., Huang, L., Zhao, J., Wang, Z., Yao, W., Wu, X., Huang, J., and Bian, B. from ref. [152]	Meta-Analysis	1453 participants from 27 case-controlled studies	“HF patients had lower zinc levels than the control subjects [Standardized mean difference (SMD): −0.740; 95% CI: −0.987, −0.493]. Subgroup analysis stratified by different geographic locations found that HF patients had lower zinc levels than the control subjects [Europe: SMD: −0.832 and 95% CI: −1.119, −0.545; Asia: SMD: −0.408 and 95% CI: −0.761, −0.055; America: SMD: −1.920 and 95% CI: −2.456, −1.384] (Figure 2 in ref. [152]). In addition, the subgroup analysis stratified by HF subgroups found that patients with idiopathic dilated cardiomyopathy (IDCM) [SMD: −0.562; 95% CI: −0.804, −0.320] and other HF patients [SMD: −0.924; 95% CI: −1.267, −0.581] had lower zinc levels than the control subjects, except for Ischemic cardiomyopathy (ICM) patients [SMD: −0.577; 95% CI: −1.353, 0.199] (Figure 3 in ref. [152]). The results showed a high statistical heterogeneity (I^2^ = 76.5%; *p* < 0.001). Publication bias was measured by using Begg’s test, which showed no evidence of significant publication bias (*p* = 0.868).”	Studies assessed observational, most individual studies relatively small, heterogeneity between studies was considerable
Kunutsor, S. K., and Laukkanen, J. A. from ref. [156]	Cohort	1652 men aged 42–61 years without a known history of hypertension in the Kuopio Ischemic Heart Disease cohort study	“During a median follow-up of 24.7 years, 259 participants developed hypertension. Serum zinc was weakly correlated with several risk markers for hypertension and nonlinearly associated with incident hypertension. In analyses adjusted for age, the hazard ratio (95% CIs) for hypertension in a comparison of the top quartile vs. bottom quartiles 1–3 of zinc concentration was 1.65 (1.27–2.15; *p* < 0.001), which was minimally attenuated on adjustment for several established risk factors 1.48 (1.13–1.93; *p* = 0.004). The association remained unchanged on further adjustment for renal function, socioeconomic status, and dietary factors. The findings were generally consistent across several clinical subgroups.”	Observational study, population restricted to patients in the Kuopio region of eastern Finland
**Copper**				
**Study/Authors/Year**	**Study Type**	**Study population**	**Findings**	**Limitations**
Kok, F.J.; van Duijn, C.M.; Hofman, a.; van Der Voet, G.B.; de Wolff, F.A.; Paays, C.H.C.; Valkenburg, H.A from ref. [162]	Case-Control	62 patients with cardiovascular death and 124 matched controls	“The adjusted relative risk of death from cardiovascular disease was 3.5 [CI: 1.4–8.7] for patients in the highest copper quintile, and 2.2 [CI: 0.8–6.4] in the lowest copper quintile.”	Observational study, small sample size, restricted to patients from Zoetermeer, The Netherlands
Wilson Tang, W., Wu, Y., Hartiala, J., Fan, Y., Stewart, A. F., Roberts, R., McPherson, R., Fox, P. L., Allayee, H., and Hazen, S. L. from ref. [163]	Cohort	4177 patients undergoing elective coronary angiography (age 63 ± 11 years, 66% male, 32% history of MI, 31% diabetes mellitus)	“Mean Copper level was 24 ± 6 mg/dL. Serum Cu level was associated with greater risk of MI at 3 years (hazard ratio [quartile 4 versus 1] 2.35, 95% confidence interval [CI] 1.79–3.09, *p* < 0.001). After adjustment for traditional risk factors, high-sensitivity C-reactive protein, and creatinine clearance, Cu remained independently predictive of major adverse cardiovascular event (MACE) (hazard ratio 1.55, 95% CI 1.10–2.17, *p* = 0.012).”	Observational study, population restricted to Cleveland Clinic GeneBank study population, population skewed towards patients with CAD who are being treated with medications to lower risk of major adverse cardiovascular events
Ford, E.S. from ref. [165]	Cohort	4574 NHANES II participants 30+ (including 764 patients who died and 151 who died from coronary artery disease)	“At baseline, the age-adjusted serum copper concentration was about 5% higher among participants who died from coronary heart disease than among those who did not (*p* = 0.072). After adjustment for age, sex, race, education, smoking status, systolic blood pressure, serum cholesterol, serum high density lipoprotein cholesterol, body mass index, recreational activity, nonrecreational activity, history of diabetes, and white blood cell count, the hazard ratios for death from coronary heart disease for serum copper concentrations in the second, third, and fourth quartiles (versus the first quartile) were 1.84 (95% confidence interval (Cl): 0.93, 3.66), 2.14 (95% Cl: 1.21, 3.77), and 2.87 (95% Cl: 1.57, 5.25), respectively.”	Observational study, older dataset, population restricted to US (NHANES II participants), only a single copper measurement was taken, patients not identified as deceased were assumed to be alive
Sharma, R. K., Sarkar, P. D., Paneri, S., Lohokare, R., Agrawal, T., and Manyal., R. from ref. [166]	Case-Control	50 patients diagnosed with myocardial infarction and 50 age and sex matched apparently healthy controls	“The mean levels of serum ceruloplasmin were 75.31 [+ or −] 9.61 mg/dl in cases and 43.2 [+ or −] 8.91 mg/dl in controls respectively. Ceruloplasmin levels were found to be highly significant (*p* < 0.001) in patients of Acute myocardial infarction when compared to controls.”	Observational study, small sample size, population restricted to patients in Indore, India, relationship between copper and ceruloplasmin are not related in a reliable manner in vivo.
Huang, L., Shen, R., Huang, L., Yu, J., and Rong, H. from ref. [175]	Meta-Analysis	1504 participants from 13 studies	“The pooled analysis indicated that patients with HF had higher serum copper than the control subjects [standardized mean difference (SMD), 0.982; 95% confidence interval (CI), (0.679, 1.285)]. Subgroup analysis stratified by different geographic locations found that HF patients had higher copper than the control subjects in Asia and Europe [Asia: SMD, 0.948 and 95% CI, (0.569, 1.327); Europe: SMD, 1.275 and 95% CI, (0.633, 1.917)], but not in America [America: SMD, 0.637 and 95% CI, (−0.109, 1.383)]. Additionally, subgroup analysis revealed that patients with ischemic cardiomyopathy (ICM) [SMD, 1.171; 95% CI, (0.717, 1.624)], idiopathic dilated cardiomyopathy (IDCM) [SMD, 0.569; 95% CI, (0.097, 1.042)] and other types of HF [SMD, 1.152; 95% CI, (0.594, 1.710)] all had higher copper levels than controls. Further subgroup analysis stratified by Newcastle-Ottawa Scale scores also found higher serum copper in patients with HF than controls within each subgroup.”	Studies included were observational, Individuals studies included all had relatively small sample sizes, significant heterogeneity between studies

## Abbreviations

Ap-1	activator protein 1
As	Arsenic
AV	atrioventricular
Ba	Barium
BFD	Blackfoot disease
BKMR	Bayesian Kernel Machine Regression
CAD	coronary artery disease
Cd	Cadmium
CHD	congenital heart disease
CI	confidence interval
Co	Cobalt
Cr	Chromium
Cu	Copper
CVD	cardiovascular disease
COMT	catecholamine-O-methyltransferase
CYP450	cytochrome p450
DM	diabetes mellitus
DMT	divalent metal transporter
DNA	deoxyribonucleic acid
ECG	electrocardiogram
EGF	epidermal growth factor
EPA	United States Environmental Protection Agency
ET-1	endothelin 1
Fe	Iron
GTF	glucose tolerance factor
HDL	high-density lipoprotein
HF	heart failure
Hg	Mercury
HOMA-IR	homeostatic model assessment insulin resistance
HTN	hypertension
ICM	ischemic cardiomyopathy
IDCM	idiopathic dilated cardiomyopathy
IHD	ischemic heart disease
JNK	c-Jun N-terminal kinase
LDL	low-density lipoprotein
LF	low frequency
LOX-1	Oxidized low-density lipoprotein receptor 1
MACE	major adverse cardiovascular event
MAPK38	p38 mitogen-activated protein kinase
MeHg	methylmercury
mRNA	messenger ribonucleic acid
NF-kB	nuclear factor-kB
NHANES	National Health and Nutrition Examination Survey
Ni	Nickel
NK-1	neurokinin 1
NO	nitric oxide
NOS	nitric oxide synthase
Nox2	nicotinamide adenine dinucleotide phosphate oxidase 2
OR	Odds Ratio
OSHA	Occupational Safety and Health Administration
PAD	peripheral artery disease
Pb	Lead
PKC	protein kinase C
PM	particular matter
RAAS	renin–angiotensinogen–aldosterone
ROS	reactive oxygen species
SDNN	standard deviation of RR intervals
Se	Selenium
SERCA2	sarcoplasmic/endoplasmic reticulum Ca-ATPase 2
SES	socioeconomic status
SMD	standard mean difference
TNF-α	tumor necrosis factor-α
TPN	total parenteral nutrition
VCAM-1	vascular cell adhesion protein 1
VLDL	very-low-density lipoprotein
Zn	zinc
